# The PTP1B inhibitor MSI-1436 ameliorates liver insulin sensitivity by modulating autophagy, ER stress and systemic inflammation in Equine metabolic syndrome affected horses

**DOI:** 10.3389/fendo.2023.1149610

**Published:** 2023-03-20

**Authors:** Lynda Bourebaba, Anna Serwotka-Suszczak, Ariadna Pielok, Mateusz Sikora, Malwina Mularczyk, Krzysztof Marycz

**Affiliations:** ^1^ Department of Experimental Biology, Faculty of Biology and Animal Science, Wrocław University of Environmental and Life Sciences, Wrocław, Poland; ^2^ International Institute of Translational Medicine, Wisznia Mała, Poland; ^3^ Department of Medicine and Epidemiology, School of Veterinary Medicine, University of California, Davis, Davis, CA, United States

**Keywords:** EMS, PTP1B, MSI-1436, liver metabolism, autophagy, insulin, inflammation

## Abstract

**Background:**

Equine metabolic syndrome (EMS) is a multifactorial pathology gathering insulin resistance, low-grade inflammation and past or chronic laminitis. Among the several molecular mechanisms underlying EMS pathogenesis, increased negative insulin signalling regulation mediated by protein tyrosine phosphatase 1 B (PTP1B) has emerged as a critical axis in the development of liver insulin resistance and general metabolic distress associated to increased ER stress, inflammation and disrupted autophagy. Thus, the use of PTP1B selective inhibitors such as MSI-1436 might be considered as a golden therapeutic tool for the proper management of EMS and associated conditions. Therefore, the present investigation aimed at verifying the clinical efficacy of MSI-1436 systemic administration on liver metabolic balance, insulin sensitivity and inflammatory status in EMS affected horses. Moreover, the impact of MSI-1436 treatment on liver autophagy machinery and associated ER stress in liver tissue has been analysed.

**Methods:**

Liver explants isolated from healthy and EMS horses have been treated with MSI-1436 prior to gene and protein expression analysis of main markers mediating ER stress, mitophagy and autophagy. Furthermore, EMS horses have been intravenously treated with a single dose of MSI-1436, and evaluated for their metabolic and inflammatory status.

**Results:**

Clinical application of MSI-1436 to EMS horses restored proper adiponectin levels and attenuated the typical hyperinsulinemia and hyperglycemia. Moreover, administration of MSI-1436 further reduced the circulating levels of key pro-inflammatory mediators including IL-1β, TNF-α and TGF-β and triggered the Tregs cells activation. At the molecular level, PTP1B inhibition resulted in a noticeable mitigation of liver ER stress, improvement of mitochondrial dynamics and consequently, a regulation of autophagic response. Similarly, short-term ex vivo treatment of EMS liver explants with trodusquemine (MSI-1436) substantially enhanced autophagy by upregulating the levels of HSC70 and Beclin-1 at both mRNA and protein level. Moreover, the PTP1B inhibitor potentiated mitophagy and associated expression of MFN2 and PINK1. Interestingly, inhibition of PTP1B resulted in potent attenuation of ER stress key mediators’ expression namely, CHOP, ATF6, HSPA5 and XBP1.

**Conclusion:**

Presented findings shed for the first time promising new insights in the development of an MSI-1436-based therapy for proper equine metabolic syndrome intervention and may additionally find potential translational application to human metabolic syndrome treatment.

## 1 Introduction

Protein tyrosine phosphatases (PTPs) constitute an important, heterogeneous group of enzymes, the activity of which is opposed to that of protein tyrosine kinases. The mechanism of action of PTP enzymes relies on the separation of phosphate groups from the tyrosine residues of various enzyme proteins, thus leading to their inactivation, which in the case of enzymes being part of the cascade, leads to the inhibition of signal transmission. One of the most common and best described PTP is phosphatase 1B (PTP-1B), which is involved in regulating the insulin receptor (IR) activity. Under the influence of primed IR- PTP-1B is also phosphorylated, and thus activated, by attaching phosphate residues to tyrosine at sites 66, 152 and 15. Active PTP-1B binds directly to the IR and dephosphorylates its tyrosine residues, leading to its inactivation. Studies have shown that PTP regulation of the insulin pathway is mediated not only by IR, but also IR substrates (e.g., IRS-1) that are deactivated through PTP-1B-mediated dephosphorylation (1–8). Dephosphorylation of the insulin receptor and its substrates leads to the inhibition of the molecular cascades stimulated by IR activation and thus suppresses the metabolic effects of insulin. Thus, the excessive activity of protein tyrosine phosphatases may be one of the causes underlying insulin resistance onset. For this reason, new methods are being sought to inhibit the activity of the PTP-1B enzyme, which seems to be one of the key factors in regulating insulin signalling and sensitivity of peripheral tissues.

In recent years, Trodusquemine commonly known as MSI-1436 has been extensively reported as a potent selective PTP1B inhibitor. In a mouse model of genetic obesity, it has been shown to cause rapid and reversible significant weight loss. Therefore it appears that, due to its ability to improve glucose tolerance, insulin sensitivity and enhance body weight loss, it is a potential anti-diabetic agent (9).

Therefore, the major goal of the presented research was to investigate the influence of MSI-1436, and thus PTP1B inhibition, on the metabolic condition of horses diagnosed with equine metabolic syndrome (EMS), an endocrine disorder that originates from improper insulin management. The disease is diagnosed more and more frequently and seems to be related to a lack of physical activity and improper diet, especially carbohydrates overfeeding. Clinical symptoms of the disease lead primarily to the development of insulin resistance, hyperinsulinemia, laminitis, hyperlipidaemia, local and systemic inflammation (10, 11). The increasing frequency of diagnosing this disease necessitates a deeper understanding of both the underlying mechanisms and the search for new diagnostic and therapeutic solutions. One of the main insulin resistance hallmarks in EMS lies in the profound deregulation of the liver metabolism. EMS liver has thus been found to display increased fibrosis caused by excessive production of extracellular matrix proteins and their insufficient degradation by metalloproteinases, inflammation related to the release of pro-inflammatory factors by damaged hepatocytes and the recruitment and activation of immune cells, which is also significant in the progressive state of organ degradation (12–14). The inability of hepatocytes to properly clear, eliminate and recycle damaged cellular components strongly participates in their activity and homeostasis decline. Autophagy, which refers to a highly conserved lysosomal degradation process is one of the main pathways responsible for maintaining proper metabolic balance and homeostasis (15). Earlier studies additionally evidenced critical abnormalities in autophagic events in the course of metabolic syndrome, type 2 diabetes and obesity, which has been associated to prolonged pro-inflammatory responses (16).

Low-grade inflammation is indeed considered as one of the most critical pathologic responses contributing to insulin resistance occurrence and metabolic syndrome progression. EMS horses have been reported to particularly exhibit elevated pro-inflammatory cytokines levels, including interleukin-1β (IL-1β), interleukin-6 (IL-6), tumor necrosis factor α (TNF-α), interferon γ (IFN-γ) and transforming growth factor β (TGF-β) at both tissue and systemic level, as well as impaired anti-inflammatory pathways evidenced by the suppression of anti-inflammatory mediators’ expression such as IL-10, IL-4 and IL-13 and more importantly, though a depletion of immunosuppressive cells activation, namely regulatory T cells (Tregs) (17). As a specialized lymphocytes subpopulation, CD3^+^CD4^+^CD25^hi^FoxP3^+^ Tregs are recognized as a pivotal element in the regulation and maintenance of immune system homeostasis. Tregs attenuate excessive and abnormal immune responses by repressing most of the immune cells functions such as lymphocytes, macrophages and B cells, *via* the release of potent mediators that include inter alia IL-10, IL-35, Cytotoxic T-Lymphocyte Antigen 4 (CTLA-4), Programmed death 1 (PD-1) and Inducible costimulator (ICOS) (18). Tregs abundance fluctuations have been previously correlated with various metabolic dysfunctions. Patients suffering from obesity, insulin resistance and diabetes type 2 were characterized by substantial decreased number of Treg cells and a significant drop in IL-10 systemic level. Experimental Tregs exhaustion further results in increased fasting glucose concentration, glucose intolerance and insulin desensitization of peripheral tissues (19), highlighting the obvious involvement of Tregs in the loss of metabolic homeostasis, and their paramount role as potential targets for the restoration of proper physiological integrity in patients affected with metabolic disorders.

Insofar EMS horses are characterized by persistent insulin desensitization, the consequent hyperinsulinemia exerts a negative feedback that strongly inhibits autophagic pathways (20, 21). Crosstalk between insulin resistance and autophagy has been reported in both animals and humans. Cai and colleagues (22), demonstrated that loss of autophagic Atg3 and Atg16L1 genes triggers insulin resistance, impaired glucose metabolism and collapsed mitochondrial biogenesis in murine adipose tissue. Similarly, expression of autophagy-related markers including LC3, Beclin 1, Atg5 and Atg7 was found to be decreased in obese mice (21), while obese and T2D patients livers displayed reduced mammalian target of rapamycin (mTOR) activity, and defective insulin receptor substrate 1 (IRS1) phosphorylation (23). The exact implication of autophagic system during metabolic syndrome pathogenesis is not fully elucidated, given the fact that contradictory reports pointed out the protective effect of autophagy during metabolic deviations, and thus postulated that autophagy maybe by contrast to other findings, upregulated during insulin resistance and inflammation (23). Öst et al. (23), emphasized that T2D and obese patients with defective insulin signal transduction were characterized by malfunctioning mitochondria and overactivated autophagy/mitophagy accompanied with increased cytosolic lipid droplets and autophagosomes formation, which subsequently contributed to the excessive release of fatty acids and ultimately the exacerbation of insulin resistance, confirming the existence of a more complex crosstalk between metabolic disorders progression and autophagic pathways.

In the current research, we aimed to investigate the clinical influence of the selected PTP1B inhibitor MSI-1436 on insulin sensitivity, inflammation and autophagy process taking place in the EMS liver. Thus, impact of PTP1B inhibition using MSI-1436 on liver ER stress, mitochondrial dynamics and autophagic network has been evaluated in an *ex vivo* model of EMS liver explants, and following clinical application of the inhibitor on diagnosed EMS horses.

## 2 Materials and methods

### 2.1 Ethical approval

This study was approved by the Local Ethics Committee for Animal Experiments in Wrocław (Resolution no. 058/2021/P1 of 23.09.2021 and resolution annex no. 035/2022/NZP/DO of 20.07.2022).

### 2.2 Animals’ qualification

Liver tissue samples were collected post-mortem at a local slaughterhouse, from fasting 27 horses of both genders and various breeds at early morning. Animals qualified for this study were assigned to either EMS or Healthy group. Horses were chosen based on qualification criteria such as body weight (BW), body condition score (BCS), cresty neck score (CNS), fasting insulin levels and oral glucose tolerance test (24).

Furthermore, 6 healthy and 12 EMS Standard breed horses of various ages and gender aged between 8 and 12 years old were qualified for the MSI-1436(Trodusquemine) treatment and subsequently divided into 3 experimental groups: Healthy, EMS (a placebo group receiving saline) and MSI (horses receiving MSI-1436, Trodusquemine). The qualification was based on an extensive interview with the owner, previous history of laminitis, body weight (BW), body condition score (BCS), cresty neck score (CNS), fasting insulin concentration, and the combined glucose-insulin test (24). To further confirm hepatic insulin resistance in collected liver samples, ELISA Assays were performed for GGTP and AST concentration before and after each corresponding treatment. The qualification process was performed by an experienced veterinarian, according to criteria established in 2010 by the American College of Veterinary. Body weight assessment was performed using a mobile Bosh Equine electronic scale. Animals qualified for the EMS group were given a singular intravenous injection (vena jugularis externa) of saline while horses in the MSI group received a singular intravenous injection of MSI-1436(Trodusquemine) at the dose of 25 μg/kg b.w. One month after the treatment, blood samples and liver biopsies were collected from all of the fasting animals, according to the universal veterinary standards, as described by Rendle (25) at early morniong between 7 to 9am.

### 2.3 Liver explants

Liver tissue samples were collected post-mortem as described above, transferred into Transport Medium- Dulbecco’s Modified Eagle Medium- low glucose (DMEM-LG) supplemented with 1% penicillin and streptomycin (PS), (Sigma-Aldrich) and immediately transported to the laboratory. Tissues were washed 3 times with Dulbecco’s Phosphate Buffered Saline (Sigma-Aldrich) and placed in either standard culture medium (DMEM- LG with 1% PS, Sigma-Aldrich) or a culture medium supplemented with MSI-1436 (DMEM-LG supplemented with 1% PS and 1 μM MSI-1436, Sigma-Aldrich). After 24h of culture, tissues were fixed for RT-qPCR and protein expression analysis.

### 2.4 Determination of circulating cytokines, adipokines and insulin levels

Fresh blood samples were collected into anticoagulants-free vacutainers from each experimental group, incubated at room temperature for 30 min for blood clotting and centrifuged for 10 min at 4000 rpm in 4°C. Resulting serra were transferred to sterile polypropylene tubes, and kept at -80°C until use.

Inflammatory markers (IL-1β, TNF-α, TGF-β), adipokines (Adiponectin, Leptin) and insulin levels were measured in collected serra using specific Horse ELISA kits (**Table 1**) according to the manufacturer’s instructions. Briefly, proper volumes of serra samples and each provided standards at various concentrations were introduced into microtiter wells, and mixed with Horse antibodies specific to each targeted protein and streptavidin-HRP. The plates were incubated for 60 minutes at 37°C. Then, all wells were washed 5 times with the 1x Wash Buffer. Substrates were afterwards added to each well and incubated for 10 minutes at 37°C in the dark. A stop Solution was subsequently added, and absorbances were measured using a spectrophotometer (Epoch, BioTek, Bad Friedrichshall, Germany) at 450 nm. Final concentrations were derived from each constructed standard curve and data were analysed with GrapPad Prism 9.

**Table 1:** List of used ELISA assays kits.

ELISA Kit	Source	Catalogue N°
**Horse Interleukin 1 Beta**	BT LAB	E0079Ho
**Horse Tumor Necrosis Factor Alpha**	BT LAB	E0019Ho
**Horse Transforming Growth Factor Beta 1**	BT LAB	E0058Ho
**Horse Total Adiponectin**	MyBioSource	MBS018816
**Horse Leptin**	BT LAB	E0047Ho
**Horse Insulin**	MyBioSource	MBS044785

### 2.5 Complete blood count analysis

A volume of 10-mL of blood was drawn from each experimental group animals into K3-EDTA containing tubes, gently inverted 8 to 10 times in order to ensure a complete mixture of anticoagulant and blood, held on a tube rack and transferred to the appropriate laboratory facility for analysis. All samples were analysed for their contain in: White blood cells, monocytes and lymphocytes for systemic inflammation testing using an automated complete blood count analyser.

### 2.6 Flow cytometry analysis of regulatory T cells (Tregs)

Total peripheral blood mononuclear cells (PBMC) were isolated from each experimental group-derived blood using a Ficoll Histopaque^®^-1077-based density gradient centrifugation during 30 min, at 400 × g, at 25°C. Then, the PBMC-containing buffy coat layer was recovered and washed three times with HBSS. Obtained PBMC were then incubated with a mouse anti-horse CD4 (MCA1078GA, 1:200; Abd Serotec, Hercules, CA, USA), mouse anti-human CD25 conjugated with FITC (MA1-35144, 1:200; Thermo Fisher Scientific, Carlsbad, CA, USA) and anti-human Foxp3 conjugated with PE (61-5773-82, eBioscience, Thermo Fisher Scientific, Carlsbad, CA, USA), for 30 min at 4°C. Labelled cells were suspended in Phosphate-Buffered Saline (PBS) and phenotyped using a BD LSR Fortessa with FACSDiva version 9.0 flow cytometer equipped with an FCS Express 7.0 software (Bectona Dickinson, San Jose, USA). Data analysis was performed using the FlowJo software (TreeStar Inc., Ashland, OR, USA) for the determination of CD4^+^/CD25^+^/Foxp3^+^ cells population following appropriate gating.

### 2.7 Gene expression analysis

Total RNA was isolated from liver explants and biopsies with the phenol-chloroform method as described by Chomczynski et al., (26), using TRIZOL reagent and in accordance to the manufacturer’s protocol. The purity and concentration of isolated RNA was assessed using a nanospectrophotometer (Epoch, Biotek, Bad Friedrichshall, Germany) at a 260/280 wavelength. 500 ng of total RNA was subjected to genomic DNA digestion and subsequently used for cDNA synthesis using a PrimeScript™ RT Reagent Kit with a gDNA Eraser (TaKaRa, Gdańsk, Poland). The reaction was performed in a T100 Thermal Cycler (Bio-Rad, Hercules, CA, USA) according to the manufacturer’s protocol. Preceding the RT-qPCR analysis, the obtained cDNA was pre-amplified. Briefly, 20 ng of the synthesized cDNA was combined with a mixture of specific primers, nuclease-free water and SensiFAST SYBR & Fluorescein Kit (Meridian Bioscience, London, UK) and subjected to the following cycling protocol: 95 °C for 2 minutes, followed by 19 cycles at 95 °C for 5 seconds, 60,4 °C for 3 minutes and 72 °C for 5 seconds. Subsequently, the pre-amplified cDNA was diluted in 1:3 ratio with nuclease-free water and used for RT-qPCR analysis. Expression of targeted genes (**Table 2**) was analyzed using SensiFAST SYBR & Fluorescein Kit (Meridian Bioscience, London, UK) and performed in a CFX Connect™ Real-Time PCR Detection System (Bio-Rad). Final volume of 10 μl was used for the reaction with the following cycling conditions: 95 °C for 2 minutes, then 40 cycles at 95 °C for 15 seconds, next, annealing for 15 seconds, and elongation at 72 °C for 15 seconds. Additionally, to test alternative splicing of the XBP1 gene, an electrophoresis in a 2% agarose gel in TBE buffer with M50pz DNA Ladder (Blirt, Gdańsk, Poland) was performed for 1 hour at 100V. All of the obtained results were normalized to glyceraldehyde 3-phosphate dehydrogenase (GAPDH) expression. Relative expression of each gene was calculated using the 2^-ΔΔCQ^ method (27).

**Table 2:** Sequences of primers used for the RT-qPCR analysis.

Gene	Sequence	Amplicon Length	Accession number
LAMP2	F: GCACCCCTGGGAAGTTCTTA	147	XM_014729146.2
	R: ATCCAGCGAACACTCTTGGG		
LC3A	F: TACGCCTCCCAGGAAACCTT	183	XM_023626428.1
	R: GGGCAGAGTAGGCATGGTTG		
LC3B	F: TGAGGAGACACAAGGGAAGTC	122	XM_023637465.1
	R: AAGGTCTTCTCCGACGGCAT		
BECN1	F: AGAAGGTCCAGGCAGAGGCTGA	329	XM_001493225.4
	R: ACCCATCTTATTGGCCAGGGCG		
HSC70	F: GATTAACAAGAGGGCTGTCCGTC	74	AB292109.1
	R: GCCTGGGTGCTAGAAGAGAGA		
PINK1	F: GCACAATGAGCCAGGAGCTA	298	XM_014737247.2
	R: GGGGTATTCACGCGAAGGTA		
MFN2	F: AATGCCATGCTCTGGGACAA	325	XM_023635773.1
	R: CATCAGCGTCCAGGCAAAAC		
PARKIN	F: CTGGAGGATTTAGTCCCGGAGC	138	XM_005608125.3
	R: CCATGGCTGGAGTTGAACCTG		
NIX	F: CAAGGGCTTCTTTTCCGCAG	93	XM_005607693.3
	R: TGCAGGTCTAAGTGTGGTGG		
BNIP3	F: GTTCCTCTTCAGACACCCGA	242	XM_023636878.1
	R: GCTCCGATACACATCCTGCT		
AKT1	F: CCAGGCTTGTGGTTGTCATCCT	178	NM_005163.2
	R: TTCTTGAGGAGGAAGTACCGGG		
CHOP	F: AGCCAAAATCAGAGCCGGAA	272	XM_001488999.4
	R: GGGGTCAAGAGTGGTGAAGG		
HSPA5	F: CTGTAGCGTATGGTGCTGCT	122	XM_023628864.1
	R: CATGACACCTCCCACGGTTT		
IL-1β	F: AAACAGATGAAGTGCTCCTTCCAG R: TGGAGAACACCACTTGTTGCTCCA	391	NM_000576.3
TNF-α	F: AGTGACAAGCCTGTAGCCCA R: GTCTGGTAGGAGACGGCGAT	242	NM_000594.4
TGF-β	F: ATTCCTGGCGCTACCTCAGT R: GCTGGAACTGAACCCGTTGAT	197	NM_001081849.1
PERK	F: GTGACTGCAATGGACCAGGA	283	XM_023618757.1
	R: TCACGTGCTCACGAGGATATT		
ATF6	F: CAGGGTGCACTAGAACAGGG	164	XM_023640315.1
	R: AATGTGTCTCCCCTTCTGCG		
XBP1	F: TTACGCGAGAAAACTCATGGCC	281	XM_014742035.2
	R: GGGTCCAAGTTGAACAGAATGC		
GAPDH	F: GTCAGTGGTGGACCTGACCT R: CACCACCCTGTTGCTGTAGC	256	NM_001357943.2

### 2.8 Protein expression analysis

In order to analyse the protein expression, liver explants were homogenized on ice in a RIPA lysis buffer (5M NaCl, 0,5M tris-HCl at pH 8.0, 10% NP-40, 10% sodium deoxycholate, 10% SDS, H_2_O_mQ_) supplemented with a phosphatase and protease inhibitors cocktail. The lysates were centrifuged for 20 minutes at 4°C, 6000×g and the obtained supernatant was transferred to fresh tubes and stored at -20°C for further analysis. The protein concentration was determined using the Bicinchoninic acid (BCA) protein assay kit (Sigma-Aldrich) and the samples were diluted with a 4 × Laemmli loading buffer (Bio-Rad, Warszawa, Poland) at 75°C for 10 min. Following this step, the samples were subjected to an SDS–polyacrylamide gel electrophoresis in a Tris/glycine/SDS buffer at 100 V, the electrophoresis was performed in Mini PROTEAN Tetra Vertical Electrophoresis Cell (Bio-Rad, Warszawa, Poland) for 90 min. Next, the proteins were transferred from gel to the polyvinylidene difluoride (PVDF) membranes (Bio-Rad, Warszawa, Poland) with a Mini Trans-Blot^®^Cell (Bio-Rad, Warszawa, Poland) transfer apparatus in a Tris/glycine buffer/methanol at 100 V, 250 mA at 4°C for 45 min. The membranes were then blocked with a 5% non-fat milk solution in TBST. The membranes were first incubated overnight at 4°C with primary antibodies at recommended dilutions (**Table 3**) and then with an HRP-conjugated secondary antibody (dilution 1:2500 in TBST) for 1 h at a room temperature. ChemiDoc MP Imaging System (Bio-Rad, Warszawa, Poland) and Image Lab Software (Bio-Rad, Warszawa, Poland) were used to detect and quantify the chemiluminescent signals.

**Table 3:** List of the antibodies and their dilutions used for this study.

Antibody	Dilution	Manufacturer/Cat n°
MAP1LC3A Antibody - N-terminal region	1:1000	Aviva (arp51335)
Anti-LC3B antibody - Autophagosome Marker	1:500	Abcam (ab48394)
BECN1 Antibody - N-terminal region	1:500	Aviva (arp58595)
HSPA8 Antibody - N-terminal region (HSC70)	1:500	Aviva (arp48446)
PINK1 antibody	1:250	Biorbyt (orb331223)
Parkin Antibody (JF82-09)	1:250	Novus Biologicals (NBP2-67017)
AKT Pan Polyclonal Antibody	1:1000	Invitrogen (44-609G)
AKT (phospho-S473) antibody	1:1000	Biorbyt (orb304681)
CHOP (L63F7) Mouse mAb	1:250	Cell Signaling Technology (2895T)
BiP (C50B1@) Rabbit mAb (HSPA5)	1:1000	Cell Signaling Technology (3177T)

### 2.9 Statistical analysis

Each experiment was performed in at least three replicates. The differences between experimental groups were calculated with the one-way ANOVA method with Tukey’s test. GraphPad Prism 9 Software (La Jolla, CA, USA) was used for all statistical analyses. Differences with probability of p < 0.05 were indicated with an asterisk (*), those with p < 0.001 were showcased with two asterisks (**), differences with p < 0.001 were marked with three asterisks (***), and differences with p < 0.0001 were marked with four asterisks (****).

## 3 Results

### 3.1 The MSI-1436 regulates the CHOP-HSPA5 axis, warranting activation of transcripts essential for the elimination of ER stress effects

**Figure 1:**
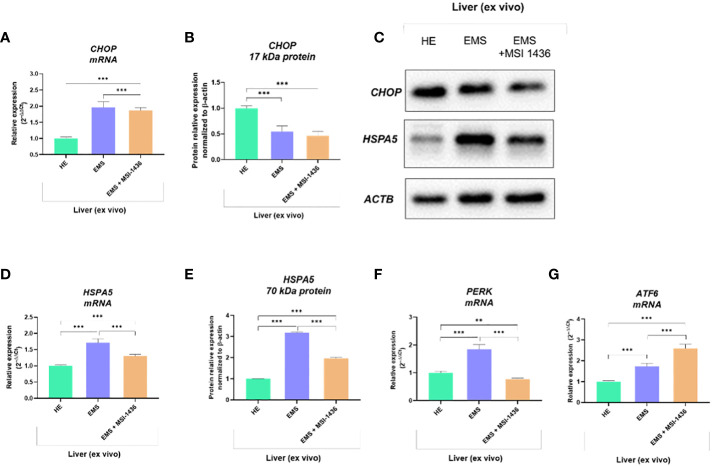
The expression pattern of ER stress-related biomarkers. The analysis included the determination of CHOP and HSPA5 expression at mRNA and protein levels **(A-E)**, and the determination of transcript levels for *PERK* **(F)** and *ATF6* **(G)**. All results are shown as mean ± SD. Columns with bars represent means ± SD. **p-value < 0.001; ***p-value < 0.001.

Expression pattern of markers associated with ER-stress, including master regulator CCAAT-enhancer-binding protein homologous protein - CHOP and its critical determinant Heat shock 70 kDa protein 5 - HSPA5/BiP, indicated on dynamics in the axis at mRNA and protein level. In this experimental setup, mRNA levels for *CHOP* were decreased in livers derived from healthy horses, while protein levels were increased (**Figures 1A–C**). The differences were significant compared to *CHOP* transcript levels and protein determined in the livers of EMS horses. The MSI-1436 reduced CHOP expression in EMS livers, but a significant difference was noted only at the mRNA level. However, the HSPA5 expression profile established on mRNA and protein levels consistently indicated its upregulation in the EMS liver (**Figures 1D, E**). At the same time, treatment with MSI-1436 caused a decline in the accumulation of HSPA5 transcript and protein in the livers of horses affected by EMS. The mRNA expression profile for *HSPA5* correlated with the Protein kinase R (PKR)-like endoplasmic reticulum kinase -*PERK* transcript levels, a major ER stress sensor that modulates global protein synthesis and participates in proapoptotic signalling. However, with the exception that MSI-1436 significantly decreased mRNA expression of *PERK* in EMS livers, both compared to untreated EMS as well as to healthy tissue (**Figure 1F**). Moreover, we noticed that treatment of EMS livers with MSI-1436 triggered an accumulation of Activating transcription factor 6 - *ATF6* transcripts, which are known to critically regulate cellular and ER chaperones genes expression. Furthermore, a comparison of *ATF6* mRNA expression showed its downregulation in control livers (**Figure 1G**).

**Figure 2:**
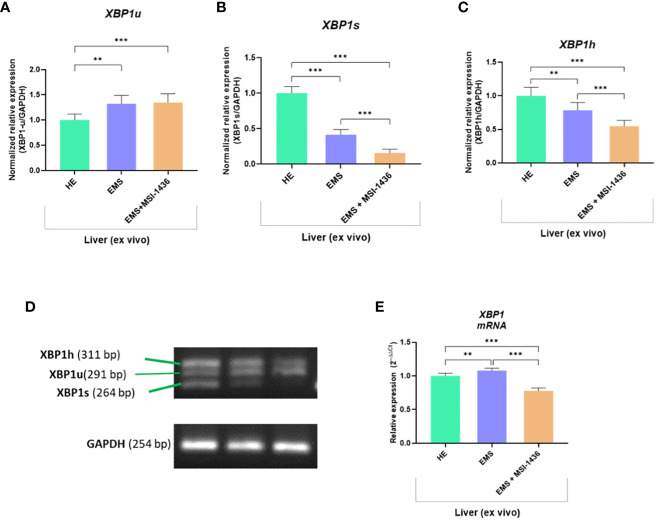
The expression of *XBP1* and analysis of its splicing. The densitometric analysis of single-stranded cDNA annealed to double-stranded *XBP1* unspliced (*XBP1*u; **A, D**) and spliced *XBP1* (*XPB1s*; **B, D**), as well as hybrid (*XBP1h*) formed from one strand XBP1u and one strand *XBP1s* **(C, D)**. The number of accumulated XBP1 transcripts was determined using RT-qPCR **(E)**. All results are shown as mean ± SD. Columns with bars represent means ± SD. **p<0.01; ***p-value < 0.001.

To further substantiate the influence of MSI-1436 application on ER stress regulation, the expression and splicing patterns of X-box-binding protein 1 - *XBP1* has been evaluated. Indeed, the *XBP1* splicing has been shown to maintain the activity of its protein in regulating the transcription of ER stress proteins that promote the degradation of misfolded proteins for proteostasis restoration. Herein, we noted that *XBP1* splicing (*XBP1s*) was decreased due to MSI-1436 treatment (**Figures 2B, D**). However, we indicated an increased accumulation of *XBP1s* in livers from control horses compared to EMS horses. Furthermore, the expression of hybrid transcripts [formed from one strand *XBP1u* (**Figure 2A**) and one strand *XBP1s* (**Figure 2B**)] was also increased in healthy horses compared to EMS horses (**Figure 2C**). Nevertheless, the real-time analysis of transcripts number for *XBP1* indicated the opposite profile, i.e., significantly decreased expression of *XBP1* in livers from control horses and increased from EMS horses. Simultaneously, real-time analysis confirmed that MSI-1436 reduce mRNA levels of *XBP1* in EMS livers (**Figure 2E**).

### 3.2 MSI-1436 facilitates autophagy in EMS-affected liver *via* upregulation of BECN1

The analysis of autophagy and mitophagy-related factors determined on livers explants indicated that MSI-1436 modulates autophagic pathways and may act at different levels, i.e., from autophagosome formation to autophagosome/endosome maturation inducing expression of Beclin-1 (*BECN1*).

**Figure 3:**
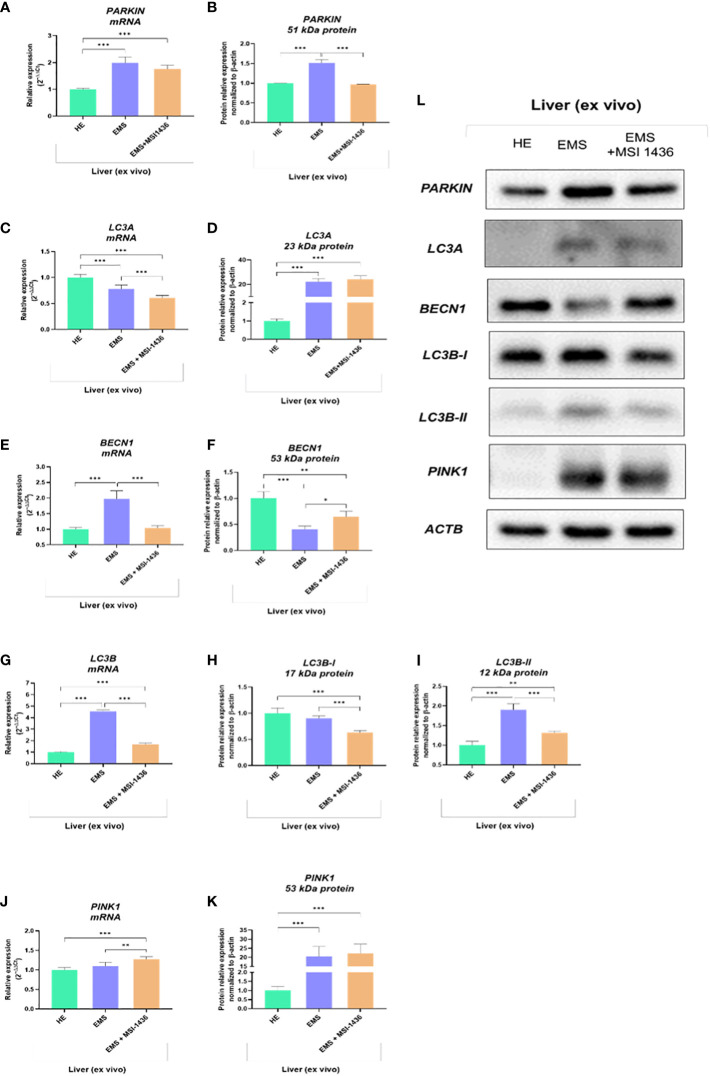
The expression of autophagy-related transcripts and proteins. The tested profile included evaluation of PARKIN1 **(A, B, L)**, LC3A **(C, D, L)**, BECN1 **(E, F, L)**, LC3B **(G-I, L)** and PINK1 **(J-L)**. Data are presented as a mean value obtained from measurments ± SD. Statistical significance was marked as follows: *p-value < 0.05, **p-value < 0.01 and ***p-value < 0.001.

The Parkin RBR E3 ubiquitin-protein ligase - PARKIN expression was increased in EMS livers compared to healthy livers at both mRNA and protein levels. The MSI-1436 did not alter *PARKIN* transcript levels but significantly reduced the intracellular accumulation of its protein (**Figures 3A, B, L**). In turn, Microtubule-associated protein light chain 3A - *LC3A* transcripts were downregulated in EMS-affected livers, while MSI-1436 only deepened the reduction in mRNA expression for *LC3A* (**Figures 3C, D**). The obtained data indicate that MSI-1436 may activate autophagy and ameliorate stress inducers contributing to increased expression of Beclin-1 at the protein level, simultaneously reducing its transcripts abundance (**Figures 3E, F, L**). The study also showed decreased mRNA expression for PTEN-induced kinase 1 - *PINK* in healthy liver tissues. Simultaneously, the accumulation of *PINK1* transcripts increased in EMS liver and was additionally upregulated by MSI-1436 (**Figure 3J**). At the same time, MSI-1436 did not alter PINK intracellular protein expression in the EMS liver (**Figure 3K**). Furthermore, *LC3B* transcripts were decreased in control livers while increased in EMS-affected tissue. The MSI-1436 significantly reduced the expression of mRNA for *LC3B*, which correlates with lowered protein expression of cytosolic LC3B-I and lipidated LC3B-II isoform (**Figures 3G–I**).

**Figure 4:**
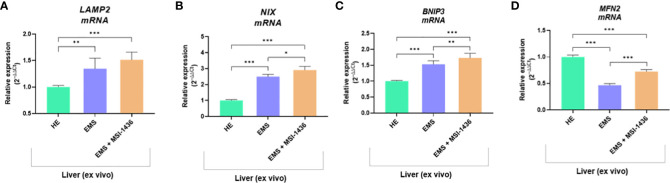
The transcripts levels associated with mitophagy determined with RT-qPCR. The comparative analysis included an evaluation of mRNA expression for *LAMP2* **(A)**, *NIX* **(B)**, *BNIP3* **(C)** and *MNF2* **(D)**. Data are presented as a mean value obtained from measurments ± SD. Statistical significance was marked as follows: *p-value < 0.05, **p-value < 0.01 and ***p-value < 0.001.

The beclin-1 (*BECN-1*) expression profile modulated by MSI-1439 correlates with mRNA levels for mitofusin-2 - *MNF-2*, which may be essential for mitophagy induction. Simultaneously, we have noted that MSI-1439 elevates the transcripts for Lysosomal Associated Membrane Protein 2 - *LAMP2*, BCL2 Interacting Protein 3 Like - *NIX* and BCL2 and adenovirus E1B 19-kDa-interacting protein 3 - *BNIP3*, indicating its potential function in mitochondria clearance and cellular homeostasis (**Figure 4**).

### 3.3 MSI-1436 activates AKT1 in livers affected by EMS

**Figure 5:**
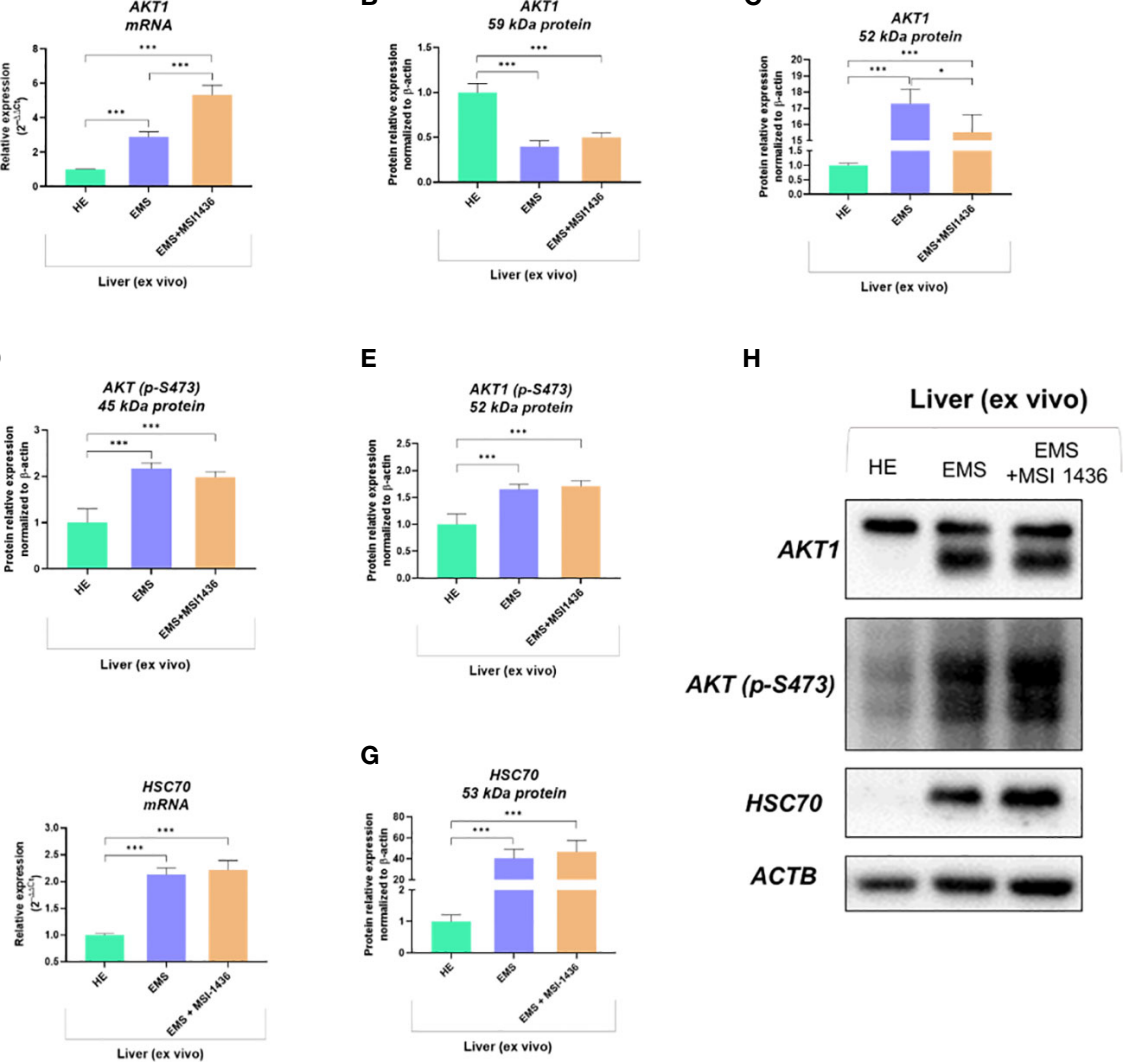
The expression of AKT1 and HSC70 governed by MSI-1436. The analysis included the determination of total *AKT1* mRNA levels **(A)**, protein expression and phosphorylation **(B-E, H)** as well as evaluation of *HSC70* transcript **(F)** and protein accumulation **(G, H)**. Data are presented as a mean value obtained from measurments ± SD. Statistical significance was marked as follows: *p-value < 0.05 and ***p-value < 0.001.

We showed that autophagy in EMS-affected liver is additionally triggered by MSI-1436 and might be associated with the activation of Protein kinase B - AKT1 and its phosphorylation. Indeed, mRNA expression for *AKT1* was elevated in EMS liver compared to control tissue, and MSI-1436 was upregulating it further (**Figure 5A**). However, we have noted a significantly increased accumulation of 59kDa AKT1 protein in healthy liver (**Figure 5B**) simultaneously with a low expression of the 52 kDa AKT1 subunit. The levels of 52 kDa AKT1 were increased in EMS-affected livers but decreased after MSI-1436 treatment (**Figure 5C**). The EMS-affected tissues had heightened levels of phosphorylated AKT1 on serine residue at position 473 (S473), which can be connected with increased AKT1 catalytic activity (**Figures 5D, E**). Nevertheless, MSI-1436 in the EMS liver did not alter the levels of activated AKT1. Moreover, increased expression of the protein folding and autophagy chaperone Heat shock cognate 71 kDa protein - HSC70 was noted in EMS liver. The MSI-1436 had a minor effect on both mRNA and protein levels - the increase of HSC70 in EMS livers treated with MSI-1435 was insignificant (**Figures 5F–H**).

### 3.4 *In vivo* MSI-1436 treatment ameliorates metabolic imbalance in EMS affected horses

**Figure 6:**
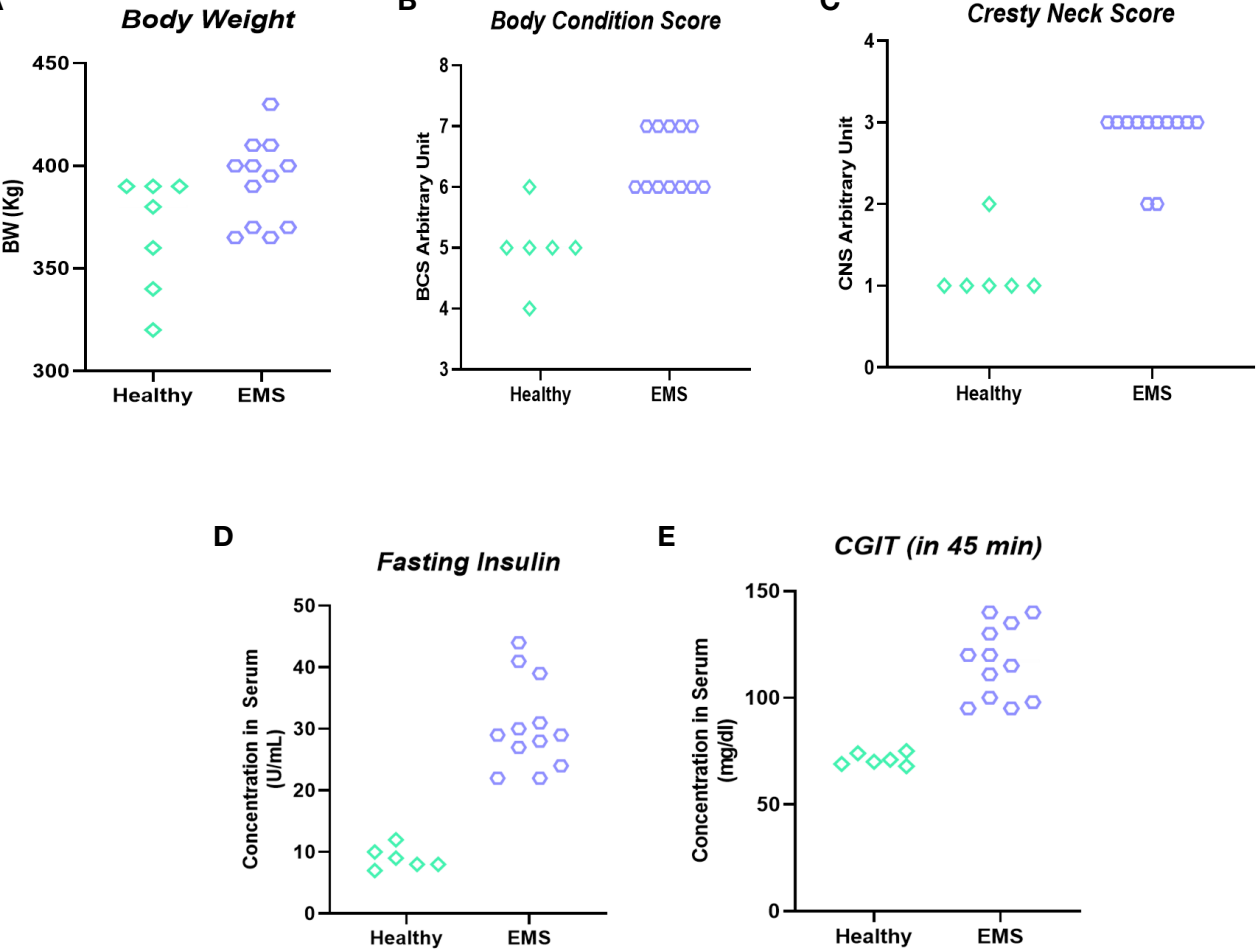
Clinical parameters used for horses’ enrolment into “experimental EMS” and “control” group. Individual data representation of body weight **(A)**, body score condition **(B)**, cresty neck score **(C)**, fasting insulin **(D)** and CGIT **(E)**.

Qualification of horses for *in vivo* studies was performed based on a system proposed by Henneke et al. (24), which warranted proper classification of animals in the experimental groups (**Figure 6**). The mean BSC index for healthy horses was 5.4 ± 0.51, while BSC for horses with equine metabolic syndrome was established at 6.1 ± 0.32 level. Similarly, the cresty neck score (CNS) which refers to the degree of adiposity was estimated at 1.4 ± 0.51 for horses considered as healthy, while in EMS group the CNS was rated at 2.8 ± 0.42 i.e., two times higher than that of healthy horses.

**Figure 7:**
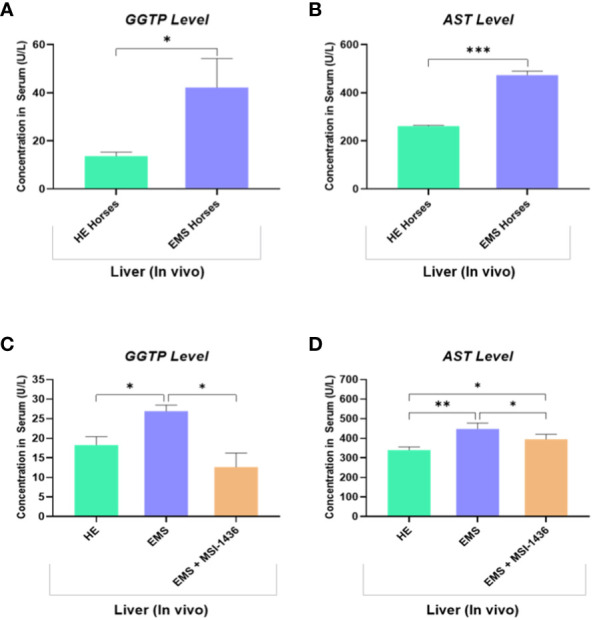
Liver function evaluation in healthy and EMS horses. Bar charts depicting the serum levels of GGTP **(A)** and AST **(B)** measured in healthy and EMS horses before treatments. Bar charts depicting the serum levels of GGTP **(C)** and AST **(D)** measured in healthy and EMS horses following 4 weeks treatment with MSI-1436 or Placebo. Data are presented as a mean value obtained from measurments ± SD. Statistical significance was marked as follows: *p-value < 0.05; **p < 0.01 and ***p-value < 0.001.

The levels of circulating biomarkers, i.e., gamma-glutamyl transferase (GGT) and aspartate aminotransferase (AST), confirmed the proper classification of experimental animals. GGT and AST range values were increased in animals assigned to the EMS group, indicating liver dysfunction by opposition to healthy horses (**Figures 7A, B**). Therewith, treatment of EMS horses with PTP1B inhibitor (MSI-1436) resulted in a visible amelioration of general liver functions and integrity as evidenced by the normalization of both GGTP and AST levels when compared to both healthy and EMS control groups (**Figures 7C, D**).

**Figure 8:**
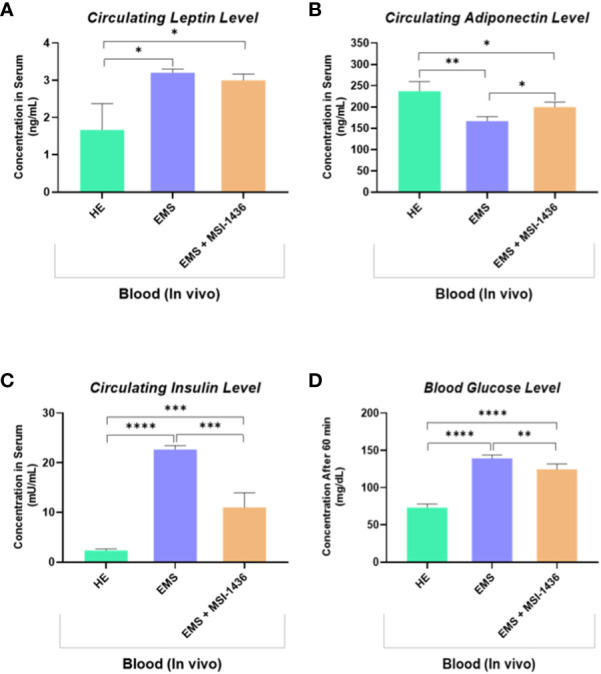
Circulating levels of key insulin resistance related metabolic parameters determined following *in vivo* application of MSI-1436 inhibitor. Blood levels of Leptin **(A)**, Adiponectin **(B)**, Insulin **(C)** and Glucose **(D)** were determined in healthy, EMS untreated and EMS treated horses. Data are presented as a mean value obtained from measurments ± SD. Statistical significance was marked as follows: *p-value < 0.05; **p < 0.01, ***p-value < 0.001 and ****p-value < 0.0001.

The impact of MSI-1436 administration on the general metabolic status of EMS horses has been further verified by measuring the blood levels of insulin, glucose and selected adipokines. As shown in the **Figures 8A, B**, EMS horses were characterized by a hyperinsulinemia concomitantly to elevated blood glucose levels when compared to healthy animals (p<0.0001). Treatment of EMS horses with the PTP1B inhibitor MSI-1436 resulted in a significant regulation of both insulinemia and glycemia (**Figures 8C, D**), which appeared lower compared to non-treated EMS animals (p<0.001; p<0.01), suggesting an amelioration of the insulin and glycaemic control and a probable attenuation of insulin resistance over a period of 4 weeks post-treatment. Furthermore, the measurement of leptin and adiponectin, two pleiotropic hormones involved in the modulation of glucose metabolism and insulin signalling evidenced a profound dysregulation in the levels of adipokines under EMS condition as compared to healthy group (p<0.05; p<0.01), which has been partly reversed in horses that received MSI-1436 in regards to the observed increased adiponectin circulating levels (p<0.05), while no significant differences in Leptin levels have been detected between EMS untreated and treated groups.

### 3.5 *In vivo* MSI-1436 application reverses liver distress in EMS affected horses

**Figure 9:**
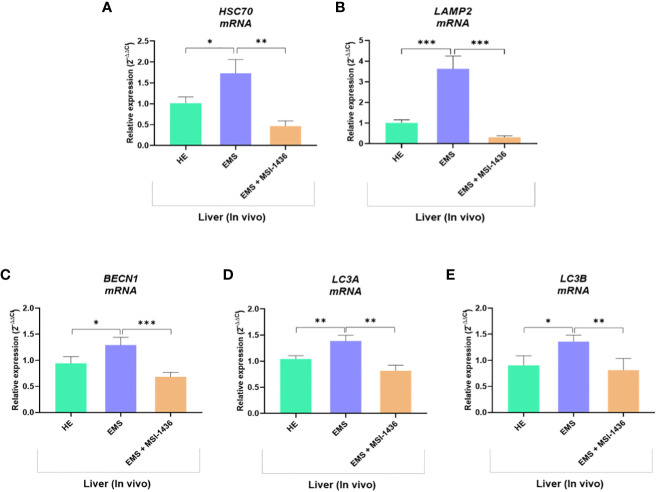
Expression of liver key autophagy associated mediators following *in vivo* application of MSI-1436 inhibitor. Relative gene expression of *HSC70* **(A)**, *LAMP2* **(B)**, *BECN1* **(C)**, *LC3A* **(D)** and *LC3B* **(E)** were determined from healthy, EMS untreated and EMS treated horses. Data are presented as a mean value obtained from measurments ± SD. Statistical significance was marked as follows: *p-value < 0.05; **p < 0.01 and ***p-value < 0.001.

We demonstrated that short-term treatment (24 h) of EMS liver explants with MSI-1436 activates the protective cellular pathways associated to autophagic system. In order to further verify how longer application of MSI-1436 to EMS horses impacts autophagic flux, gene expression of key autophagy mediators has been analysed in liver biopsies obtained from untreated and treated horses. Obtained data confirmed first that EMS horses receiving placebo exhibited increased autophagy as demonstrated by the upregulation of both *HSC70, LAMP-2, BECN1* and *LC3* by opposition to healthy horses (**Figures 9A–E**).

Interestingly, the group of EMS horses intravenously treated with MSI-1436 displayed significantly reduced autophagy one-month post-injection, by contrast to liver explants treated for a period of 24 h, in which autophagy was seen to be upregulated. Indeed, the relative expression of *HSC70*, *LAMP-2*, *BECN1* and *LC3* was found to be downregulated when compared to untreated EMS group (p < 0.001), suggesting that longer term MSI-1436 action enables to reduce liver molecular stress pathways that subsequently attenuates autophagic flux.

**Figure 10:**
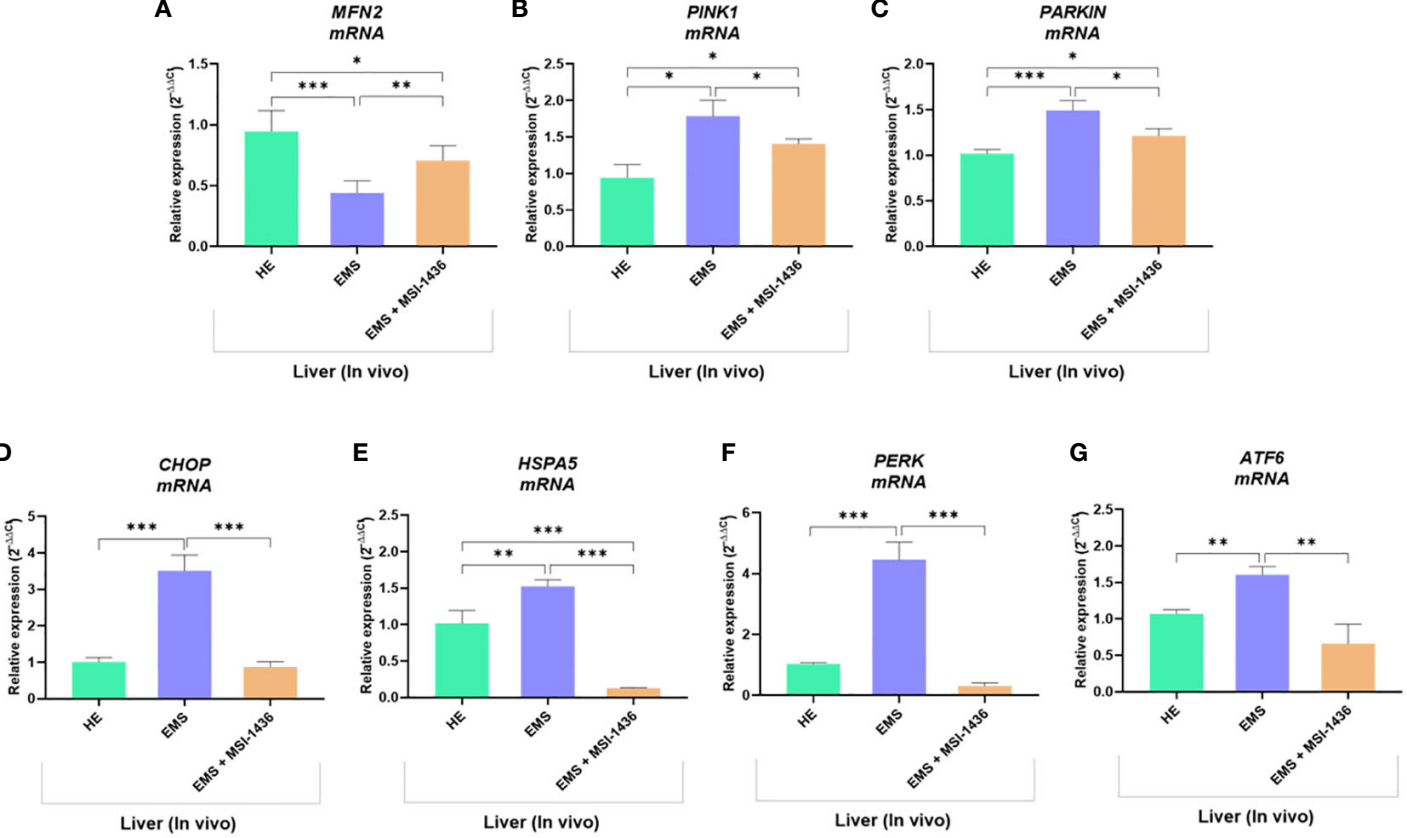
Impact of MSI-1436 administration on key mitochondrial dynamics and ER stress related markers expression in EMS affected horses. Relative gene expression of Mitochondrial dynamics **(A, B, C)** and ER stress **(D, E, F, G)** mediators analysed using RT-qPCR technique. Data are presented as a mean value obtained from measurments ± SD. Statistical significance was marked as follows: *p-value < 0.05; **p < 0.01 and ***p-value < 0.001.

To confirm this hypothesis, the expression of gene involved in the regulation of mitochondrial dynamics and ER stress was additionally analysed. As shown in **Figures 10A–C**, liver tissue derived from untreated EMS group exhibited impaired mitochondrial homeostasis and resulting dysregulation of *MFN2*, *PINK1* and *PARKIN* transcripts levels (p < 0.05; p < 0.01). Moreover, the expression of key ER stress factors namely, *CHOP, HSPA5, PERK* and *ATF6* was also found to be profoundly increased in the same experimental group (**Figures 10D–G**). Obtained data similarly evidenced the beneficial effects of MSI-1436 treatment on liver metabolic distress. Liver biopsies isolated from MSI-1436-treated EMS horses where thus characterized by reduced expression of ER stress markers (*CHOP*, *HSPA5*, *PERK* and *ATF6*) and improved mitochondrial dynamics related regulators, where relative expression of *MFN2* was restored while that of *PINK1* and *PAKIN* was downregulated when compared to untreated EMS group, suggesting that longer PTP1B inhibition using MSI-1436 abolished the various molecular stresses in EMS liver, which subsequently attenuates the increased bulk autophagic-degradation machinery.

### 3.6 *In vivo* MSI-1436 administration attenuates systemic inflammation in EMS horses

**Figure 11:**
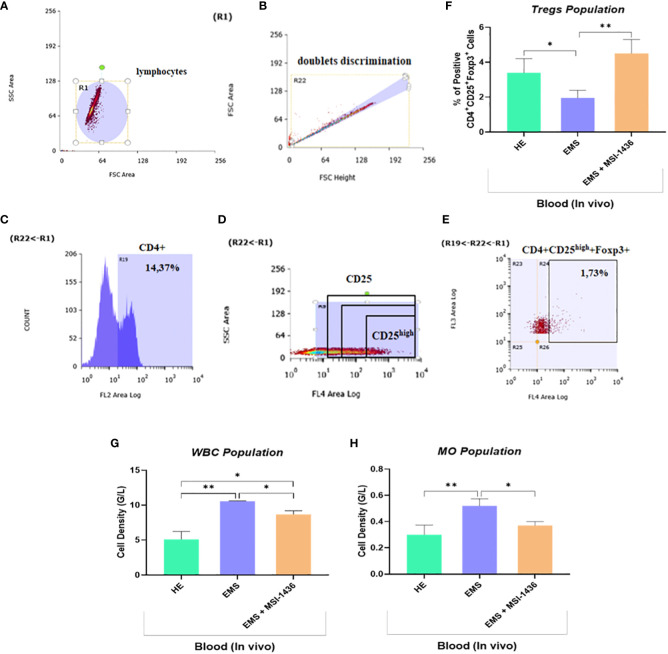
Effect of *in vivo* MSI-1436 treatment of inflammatory cells distribution in EMS horses. Gating strategy of the flow cytometry analysis of T cells in peripheral blood. R1 gate was set up around the cell population visible in the FSC/SSC axis **(A)**. The doublets were excluded from analysis by set up the gate R22 **(B)**. Gate R19 was set up on the cells positive to CD4 **(C)**. The division of CD25 on CD25 negative and positive (dim, medium and high) **(D)**. The percentage of the triple positive cells (CD4+CD25+Foxp3+) was established from set up the quadrants R24 **(D)**. Additionally, the percentage of the CD4+CD25high+Foxp3+ cells (Tregs) was established **(E)**. Average Tregs levels determined in each experimental group **(F)**. Total white blood cells (WBC) **(G)** and monocytes (MO) **(H)** densities obtained from the complete blood count analysis. Data are presented as a mean value obtained from measurments ± SD. Statistical significance was marked as follows: *p-value < 0.05 and **p < 0.01.

Low-grade inflammation represents one of the salient hallmarks of EMS condition in relation to insulin resistance, altered lipid metabolism and adipose tissue homeostasis. To this extent, the influence of prolonged PTP1B inhibition using its selective inhibitor trodusquemine on persistent inflammatory response processes has been evaluated. The complete blood count of EMS horses that received placebo demonstrated an elevation in the proportion of white blood cells and monocytes compared to healthy animals (**Figures 11G, H**). Furthermore, untreated EMS-derived PBMC displayed reduced levels of regulatory T cells (Tregs) analysed using flow cytometry (**Figures 11A–E**), as evidence of disrupted anti-inflammatory pathways (**Figure 11F**). Treatment of EMS horses with trodusquemine exerted a positive effect on systemic inflammation by lowering the total levels of lymphocytes and monocytes (p<0.05) by opposition to untreated horses. Interestingly, PTP1B inhibition enabled to augment and normalize the number of Tregs to a basal level (p<0.01), which suggests a potential regulatory effect on inflammatory responses.

**Figure 12:**
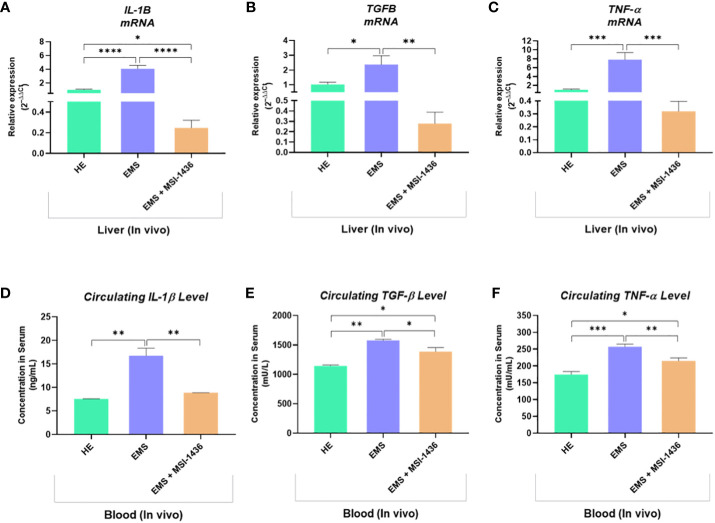
Inflammatory mediators status changes resulting from the *in vivo* MSI-1436 administration to EMS affected horses. Relative gene expression of *IL-1β* **(A)**, *TGF-β* **(B)** and *TNF-α* **(C)** determined by means of RT-qPCR technique in liver biopsies sampled following 4 weeks of MSI-1436 treatment. Circulating levels of IL-1β **(D)**, TGF-β **(E)** and TNF-α **(F)** proteins quantified using specific ELISA assays in blood samples derived from each experimental group. Data are presented as a mean value obtained from measurments ± SD. Statistical significance was marked as follows: *p-value < 0.05; **p < 0.01; ***p-value < 0.001 and ****p-value < 0.0001.

To further confirm the anti-inflammatory outcomes of PTP1B inhibition, expression levels of key inflammatory mediators have been analysed at both mRNA and protein levels. As depicted in the **Figure 12**, under EMS condition, horses were characterized by a significant upregulation of *IL-1β*, *TNF-α* and *TGF-β* transcripts in liver, as opposed to healthy animals (p<0.0001; p<0.001; p<0.05). Similarly, the plasma protein levels of the same cytokines appeared to be critically elevated by approximately 2-folds for IL-1β, 1.4-folds for TGF-β and 1.6-folds for TNF-α in regards to control horses (**Figures 12D–F**). Intravenous administration of MSI-1436 resulted in a substantial lowering of the analysed circulating pro-inflammatory cytokines, with a pronounced effect observed for IL-1β, where its level has been decreased by up to 2-folds compared to untreated EMS horses (p<0.01). Comparable trends were noted at the mRNA level, where liver expression of both *IL-1β*, *TNF-α* and *TGF-β* has been found to be considerably downregulated in comparison to placebo-treated animals (**Figures 12A–C**). Surprisingly, the MSI-1436-treated horses further displayed comparable *TNF-α* and *TGF-β* mRNA levels, and even reduced *IL-1β* transcript expression in relation to heathy group, evoking a substantial potential for PTP1B inhibition using MSI-1436 in modulating EMS-associated inflammatory bias.

## 4 Discussion

Equine metabolic syndrome (EMS) is a systematic disorder presenting a phenotype of insulin resistance, increased adiposity, and predisposition to laminitis development in equines (28, 29). Excessive accumulation of free fatty acids (FFAs) in non-adipose organs engenders cellular metabolism collapse and organelles dysfunctions, that ultimately trigger low-grade inflammation progression and associated, insulin resistance (IR) in the hepatic tissue. Critically, the hepatic IR is the primary event that subsequently leads to the progression of insulin resistance in other peripheral tissues and finally to the development of EMS (30, 31). We have previously showed that various tissues isolated from horses suffering from EMS are characterized by deteriorated autophagy, mitophagy, and endoplasmic reticulum stress (ER-stress) (32, 33). Importantly, protein–tyrosine phosphatases, such as PTP1B and LMPTP, are considered as negative regulators of insulin signalling pathways and thus might serve as promising therapeutic targets in the treatment of EMS. Previously, we have noted a positive effect of PTP1B inhibitor (Trodusquemine/MSI-1436) on EMS horse-delivered ASCs (adipose tissue-delivered stem cells) and human HepG2 cell line (34, 35). It was shown that MSI-1436 regulates autophagy and ER-stress while decreasing oxidative stress and lipotoxicity in both cellular models. To support these findings, we evaluate for the first time the therapeutic effect of PTP1B inhibitor *in vivo* and *ex vivo*, using EMS horse-derived liver tissues. The use of Trodusquemine for the proposed research was not only motivated by its proven antidiabetic and anti-obesity effects, but also by its well tolerability and well-established pharmacological profile. Indeed, various studies established the MSI-1436 safety on different animal models including mice and zebrafish, and reported effective doses being 5–50 times lower than the maximum dose tolerated by humans (9, 36–38). Trodusquemine has also been tested for its safety and pharmacokinetics in healthy human volunteers [NCT00509132, 2007]. Moreover, MSI-1436 is currently under evaluation in Phase 1 and 1b clinical trials for the treatment of obesity and type-2 diabetes [NCT00606112, 2008; NCT00806338, 2009], HER-2 positive metastatic breast cancer [NCT02524951, 2018], as well as atherosclerosis and inflammation [NCT04235023, 2020]. The data generated so far demonstrated that the PTP1B inhibitor is well tolerated by human patients and is planned to be moved to phase 2 trials (39), all of which strongly suggest its safe use for EMS intervention in veterinary clinical practice.

In this investigation, we have found that trodusquemine positively regulates autophagy and mitophagy while simultaneously modulating the ER stress and systemic insulin resistance.

It was clearly shown that MSI-1436 positively stimulates autophagy processes in isolated liver explants. It was presented by an increased accumulation of *LAMP2* and *HSC70* transcripts and a greater concentration of BECN1 and HSC70 proteins in MSI-treated tissue compared to untreated tissue. The obtained results stay in line with the data presented by other authors. Experiments on hepatocyte-specific DGAT1 knockout mice revealed that restored LAMP-2 expression in livers improved autophagy function and ameliorated alcohol-induced liver injury (40). In addition, LAMP-2 is an indispensable component of complete CMA (chaperone-mediated autophagy) that is mediated by heat shock cognate 71 kDa protein (HSC70) (41). We have noted significant discrepancies in the protein accumulation of evaluated LC3 splice variants responsible for autophagosomes and autolysosome formation. LC3A was upregulated, while LC3B-I and LC3B-II decreased after MSI-1436 treatment. Significant accumulation of complete LC3 protein is commonly associated with autophagosome formation, while downregulation of LC3B subunit seems to have no significant influence on autophagy efficiency. As reported by Baeken et al. (2020), the nuclear trapping and other methods of LC3B inhibition in human IMR90 cells are actively buffered by LC3A accumulation and compensated by LC3C (42). Longer term MSI-1436 application resulted in different effects on liver autophagy machinery. Indeed, intravenous administration of MSI-1436 to EMS horses triggered a reduction in gene expression of key autophagy mediators including *HSC70*, *LAMP-2*, *BECN1* and *LC3A/B*, suggesting a possible modulatory effect on liver tissue degradative processes. Previous reports demonstrated that various metabolic stress conditions such as lipotoxicity, insulin resistance, or inflammation upregulate autophagy owing to its cytoprotective functions and, in order to maintain cellular and tissular homeostasis by improving cell viability/death balance and attenuating the underlying inflammatory state (43, 44). Thus, the persistence of the cellular stressors including ER/Oxidative stress and inflammation sustains the transcriptional upregulation of autophagy genes, while excessive or persistent autophagy can also promote apoptosis (45).

Similarly to our findings, other studies proved that alleviation of metabolic dysfunctions-related events contributes to the attenuation of the adaptative processes constituting autophagy. Escribano-López and collaborators (46), showed that treatment of Type 2 Diabetes patients with the SS-31 Mitochondrial Antioxidant compound, reduced oxidative and ER stress, improved mitochondrial functions and decreased Beclin1, LC3 I and LC3 II expression, which attenuated autophagy response. Therewith, other research demonstrated that inhibition of PTP1B abolished ER stress-dependent autophagy activation in rats through the downregulation of Beclin-1 and LC3-II/I pathways (47). Also, PTP1B knockdown has been reported to efficiently suppress overactivation of autophagy in endothelial cells (48), as well as PTP1B deficient mice displayed reduced LC3B-II, LC3B-II/LC3B-I ratio, Atg5, Atg7 and p62 adaptor protein under tunicamycin-induced ER stress, pointing out the critical therapeutic benefice of PTP1B activity suppression (49). Taken together, it is plausible to postulate that prolonged inhibition of PTP1B with MSI-1436 in EMS horses may indirectly attenuate autophagy by reducing other metabolic stress responses including oxidative and ER stress as well as mitochondrial impairment.

Dysfunctions of mitochondria fission and fusion processes, as well as mitophagy, have been reported to play a considerable role in the development of free fatty acid-induced hepatic insulin resistance (50, 51). Mitophagy is a catabolic process that selectively degrades damaged or superfluous mitochondria. It reverses mitochondrial dysfunction and preserves mitochondrial dynamics and proper functionality. For that reason, mitophagy could promote mitochondrial fatty acid oxidation, thus inhibiting hepatic fatty acid accumulation and improving liver functionality during insulin resistance. We have shown that the PTP1B inhibitor upregulated *PINK1, MFN2, NIX* and *BNIP3* transcripts in equine liver explants. Previously, using neuronal N2a and hepatic L02 cells, it was demonstrated that PINK1 activation is crucial for PARKIN recruitment and phosphorylation, which further induce LC3-II colocalization with mitochondria and leads to mitophagy progression (52, 53). Moreover, Bnip2 and Nix are subunits of mitochondrial BNIP3L/Nix protein located in the outer membrane. This protein belongs to the BH3-only protein from the BCL2 family and thus regulates also the cell viability. BNIP3L/Nix serves as a mitophagy receptor that recognizes autophagosomes, hence participating in the selective removal of damaged mitochondria (54–56). Moreover, the overexpression of Mnf2 that regulates mitochondria fusion is essential for metabolic homeostasis of liver tissues. It has been shown that the ablation of Mfn2 in a mice model results in glucose intolerance, increased hepatic gluconeogenesis, impaired insulin signalling, as well as the development of ER stress (57).

*In vivo* application of MSI-1436 to EMS horses resulted in similar amelioration of mitochondrial dynamics. The PTP1B inhibitor exerted a strong regulatory effect on the expression of *MFN-2*, *PINK1* and *PARKIN* confirming the efficacy of the inhibitor in improving mitochondrial and overall liver metabolism under EMS condition. Previously, ablation of PTP1B in mice has also been shown to improve mitochondrial integrity by suppressing ER-stress mediated overexpression of Pink1 and Parkin (49), which reinforces the beneficial use of PTP1B inhibitors for the restoration or improvement of mitochondrial biogenesis.

It has been shown previously using a broad spectrum of liver cell lines and hepatoma cells that ER-stress impairs insulin signalling by depleting the insulin receptor on the cell surface (58). The disruption of ER homeostasis leads to ER-stress that activates unfolded protein response (UPR) (59). Here, we have shown that the use of MSI-1436 in either *ex vivo* or *in vivo* model of equine EMS has therapeutic potential for ER-stress mitigation evidenced by decreased level of transcripts commonly indicated as important ER-stress markers, i.e. *HSPA5*, *PERK* and *XBP1* (60–62). The results are consistent with other studies focused on MSI-1436 therapeutic potential in a mouse model of diet-induced obesity and IR-dependent atherosclerosis (9, 63). Abdelsalam et al. (48), reported a critical suppression of ER stress related markers CHOP, BiP, ATF-4 and GRP94 following PTP1B inhibition in endothelial cells. Furthermore, PTP1B knockout mice were found to be protected against ER stress when exposed to Tunicamycin, and further prevented the activation of TRIB3, Atg5/7, LC3B and p62 proteins while ameliorated IRS-1 tyrosine phosphorylation (49). PTP1B silencing in experimental mice similarly prevented obesity-induced ER stress by inactivating CHOP, BIP, GRP94, ATF4 and XBP1 factors (64). Interestingly, obtained results suggest a selective way of ER-stress signalling pathways regulation after PTP1B inhibition, that was evidenced by an upregulation of *ATF6* expression and confirmed by XBP1 splicing depletion.

Upon aberrant misfolded proteins accumulation, ER stress is initiated *via* a series of genes transcription and proteins expression that activate three distinct adaptative arms including the activating transcription factor 6 (ATF6) signaling, which triggers the assembly of molecule chaperones, the transcription of ER stress effectors, and mediates proper protein folding (65). Interestingly, ATF6 deficiency alters the expression of ER chaperones and exacerbates liver injury, necroptosis, impaired fatty acid oxidation, steatosis and insulin resistance arising from acute stress (66). Herein, we observed that EMS livers exhibited increased expression of *ATF6* transcript, as a result of the increased ER perturbations and metabolic stress signals load. Increased expression of *ATF6* under EMS condition is directly related to its adaptative role in the activation of molecular chaperones that sense ER stress and tend to activate homeostatic responses to protect hepatic cells from ER stress-induced damage and apoptosis (67, 68). Remarkably, treatment of EMS liver tissue with the PTP1B inhibitor MSI-1436 substantially induced *ATF6* overexpression, evoking the selective stimulation of an ATF6-protective pathway. This hypothesis is supported by previous findings showing that enhanced expression of liver endogenous chaperones including ATF6- GRP78 axis fosters the heightening of protective UPR and expression of additional chaperones such as GRP94, that cooperate to further attenuate lipids accumulation and promote their clearance from the hepatocytes in ob/ob mice (69). Consistently, *ATF6* overexpression in obese or diabetic livers regulates blood glucose level, reduces glucose intolerance and ameliorates insulin sensitivity (70, 71). Moreover, other research reported on the beneficial outcomes of ATF6 pathway stimulation in facilitating pro-survival UPR, for the prevention of diabetes progression (72). Hence, it can be concluded that MSI-1436 ameliorates liver metabolic homeostasis by targeting the folding capacity of the ER *via* an ATF6-selective pathway.

Another important UPR event is represented by the IRE1α-mediated XPB1 splicing. The dimerization and trans-autophosphorylation of IRE1α under prolonged misfolded protein burden activates its C-terminal RNase domain that catalyses the unconventional splicing of X-box–binding protein 1 mRNA. The resulting spliced *XBP1* mRNA is further translated to an active and stable form of the XBP1s protein, which initiates and regulates the expression of main UPR chaperones, Endoplasmic Reticulum-Associated Degradation (ERAD) elements and ER biogenesis mediators (73). Hither, we observed an upregulated expression of unspliced *XBP1* transcript in liver tissue derived from EMS horses. Intriguingly, the level of spliced XBP1 was found lowered compared to healthy livers, suggesting an altered UPR program. These observations can be explained on the basis of previously published research demonstrating an impaired XBP1s processing and nuclear translocation in the course of various metabolic conditions such as obesity, insulin resistance, and type 2 diabetes (74, 75). The reported progressive decline in hepatic XBP1 post-transcriptional splicing has been mainly attributed to an increase in IRE1α S-nitrosylation and depleted endoribonuclease activity mediated by elevated inducible nitric oxide synthase (iNOS) activity, during metaflammation characteristic of metabolic syndrome and obesity (76). Noteworthy, incubation of EMS liver explants with MSI-1436 resulted in a further decreased expression of total *XBP1* mRNA and spliced *XBP1* level. This outcome is in agreement with our recently published data, showing the ability of MSI-1436 to blockade *XBP1* splicing in a model pf tunicamycin-indued ER stress in HepG2 cell line (77). These findings additionally suggest that the mechanisms underlying XBP1 functional outputs under metabolic ER stress differ from other proteostatic perturbators and seemingly encompass more intricate and interconnected molecular events, and the implication of unrelated external influences leading to either increased or decreased IRE1α-XBP1 axis activation (78). However, we bring the evidence that MSI-1436 application may represent an efficient genetic inhibitor of certain aspects of IRE1α activity for the attenuation of sustained XBP1 expression and unconventional splicing under unfavourable metabolic conditions in the liver, as previously reported by Bailly-Maitre and colleagues (79), who uncovered that the inhibition of IRE1α-mediated XBP1 splicing using Bax-inhibitor 1 protects mice from obesity-associated insulin resistance development.

Unresolved or maladaptive misfolded protein burden dictates cell fate and leads to the expression of chaperone proteins that shorten cellular lifespan and initiate apoptotic cascades. Among those death signalling molecules, CHOP transcription factor is considered as a major misfolded protein stress-associated pro-apoptotic mediator. It stimulates the expression of a variety of upstream effectors including BIM, BAK, BAX, TRB3 and Caspase 3 (80). Here we found that EMS livers were characterized by upregulated *CHOP* mRNA and unexpected reduced CHOP protein level, whereas MSI-1436 treatment enabled to substantially downregulate *Chop* gene expression, without restoring normal levels of its protein. This divergence between gene and protein expression has already been previously reported in other studies, in which changes in mRNA levels initiating the UPR and ER stress have been found to not translate into protein changes. What is more, the occurrence of various post-translational modifications that fluctuate with the degree of metabolic failure have been shown to orchestrate chaperone proteins downregulation, protein synthesis and ubiquitination deterioration, as well as intracellular trafficking and excretion decline under sustained metabolic stress (81, 82), suggesting that EMS condition may disrupt the balance between gene and protein expression, and MSI-1436 might rather specifically attenuate ER stress at gene transcriptional level instead of protein post-translational maturation.

Our obtained data thus evidenced a reduced CHOP protein abundance under EMS condition, which was not restored by MSI-1436. Although CHOP protein is expected to be upregulated in the course of ER stress progression, previous investigations have highlighted a counterintuitive implication of CHOP factor in the onset of obesity and liver steatosis. Indeed, the development of obesity and associated excessive fats deposition in mice has been associated with a depletion in CHOP protein. Moreover, CHOP has been found to strongly suppress the expression of lipids metabolism master regulators including *CEBPA, PPARA*, and *SREBF1* and to consequently hamper adipogenesis and adipose tissue expansion (83–85). In the liver, CHOP protein suppression has similarly been associated with steatosis occurrence, excessive lipids accumulation within hepatocytes and generally establishment of leptin resistance (86, 87). Moreover, CHOP depletion has been shown to promote liver inflammation, impaired glucose and insulin tolerance during high fat feeding (84), suggesting the important role of CHOP protein as a molecular mediator that is altered during systemic metabolic impairments similar to EMS. Taken together, these findings substantiate the protective effect of MSI-1436 on liver metabolic distress that translates through ER stress adaptation, mitochondrial failure attenuation and subsequent autophagy regulation, which ultimately leads to a substantial amelioration of liver functions.

Liver insulin desensitization has been largely associated to increased adiposity and low-grade inflammation persistence. During metabolic syndrome progression, the highly pro-inflammatory microenvironment is typically maintained by the constant release of abnormal cytokines levels including IL-1β, TNF-α, IL-6 and, acute phase proteins, which strongly contribute to the metabolic failure of metabolically active organs such as liver (88). In this study, we found that EMS horses displayed elevated levels of IL-1β, TNF-α and TGF-β, which are known as pivotal pro-inflammatory mediators and were characterized by higher monocytes number and poor CD4+CD25+Foxp3+ regulatory T cells activation. What is more, we demonstrated that treatment of EMS horses with the potent PTP1B inhibitor MSI-1436 resulted in a significant regulation of inflammatory responses, evidenced by a substantial decreased levels of both IL-1β, TNF-α and TGF-β at mRNA and protein level, and by an interesting stimulation of CD4+CD25+Foxp3+ regulatory T cells activation. Previous investigations reported on the close crosstalk between inflammation and PTP1B activity. Song et al. (89), found that PTP1B expression increases significantly after inflammation induction using LPS, and that it potentiates the microglial proinflammatory response. Moreover, they showed that pharmacological PTP1B blockade resulted in a marked suppression of iNOS, COX-2, TNF-α, and IL-1β levels, which stands in line with our observed results. Similarly, PTP1B deficiency in a model of high fat diet-induced obesity in mice has been reported to substantially protect against hypothalamic microglia inflammation, which has been attributed to a restoration of the JAK2-STAT3 signalling, initially negatively regulated by PTP1B overactivation (90). Our data are furthermore in agreement with earlier researches that evaluated the impact of liver PTP1B inhibition or deficiency on inflammatory processes. Loss of PTP1B in the course of liver steatosis and fibrosis directly suppressed the expression of TGF-β, while its depletion in a model of ethanol-induced liver injury in mice resulted in a visible attenuation of induced injury, inflammation, and steatosis that has been correlated with a reduced oxidative stress (91, 92). Therewith, Wiede and collaborators (93), recently demonstrated similarly to our findings that global or hematopoietic deletion or inhibition of PTP1B with MSI-1436 is accompanied by the recruitment of a number of immunosuppressive cells including CD4+ regulatory T cells (Tregs) mediated by the STAT-5 signaling priming in C57BL/6 mice, highlighting the importance in modulating PTP1B for the proper control of liver deterioration associated to inflammatory pathways.

Similar outcomes have been observed regarding the levels of selected adipokines, including adiponectin, which appeared to be augmented in horses having received MSI-1436 inhibitor. As a consequence, we observed that MSI-1436-treated horses exhibited lower circulating concentrations of insulin and glucose compared to untreated animals, which displayed critical hyperinsulinemia, hyperglycaemia and hypoadiponectinemia as prominent EMS clinical manifestations. Adiponectin is among the most important hormones secreted by adipose tissue with strong insulin sensitizing properties. Moreover, the fat cytokine is known to participate in glucose metabolism regulation and to exert anti-inflammatory properties (94). Adiponectin levels have been previously reported to be substantially reduced in patients suffering from obesity, diabetes mellitus, cardiovascular diseases and metabolic syndrome, which has been further correlated to the progression of insulin resistance and chronic inflammation of the metabolic organs (95). Here we found that EMS horses exhibited critical low adiponectin levels and that PTP1B inhibition inversely correlates with higher adiponectin levels; this is in accordance with a previous study of Swarbrick and colleagues (96), who showed that specific PTP1B inhibition with ISIS-113715 inhibitor significantly increased blood adiponectin concentrations by 70% in obese and insulin-resistant rhesus monkeys. Likewise, nonspecific PTP1B inhibition in a model of Zucker diabetic fatty (ZDF) rats has been brought out to restore the plasma adiponectin levels, which were even comparable to normal rats (97).

The restoration of normal adiponectin levels may further explain the observed anti-inflammatory effect of MSI-1436, and together substantiate the visible regulation of insulinemia and glycemia in EMS horses. Indeed, consistently to our findings, increased adiponectin levels consequently to PTP1B inhibition has been correlated to an amelioration of insulin sensitivity and a resulting decreased hyperinsulinemia and glucose intolerance in various animal models (96–99). In type 2 diabetic human patients, PTP1B inhibition also resulted in a remarkable reduction in fed and fasted glucose and HbA1c levels parallelly to lowered insulin blood levels, in relation to an improved adiponectin secretion (100).

Taken together, our and others data clearly suggest the effectiveness of PTP1B inhibition in improving the metabolic balance under insulin resistance and metabolic syndrome condition. These positive outcomes essentially derive from the attenuation of various molecular pathophysiological processes including low-grade inflammation, hormonal imbalance, ER stress, mitochondrial dysfunction and autophagy that contributes to the restoration of insulin sensitivity and proper control of glucose disposal in insulin-responsive tissues.

## 5 Conclusion

In the present study, we have performed the *ex vivo* and *in vivo* assessment of the potential therapeutic efficiency of PTP1B inhibitor for the first time using the liver explants of EMS-suffering horses. The obtained results indicate that MSI-1436 might serve as an essential therapeutic agent modulating the autophagic processes of liver cells delivered from insulin-resistant horses. The addition of MSI-1436 positively regulates autophagy and autophagosome formation and induces mitophagy. At the same time, it inhibits the ER-stress resulting from ongoing inflammation due to pathological deposition of free fatty acids within the non-adipogenous organs. *In vivo* administration of MSI-1436 to EMS horses further confirmed its potent therapeutic effect on rescuing distressed liver and restoring proper metabolic homeostasis, by reducing inflammatory processes, and regulating the circulating levels of insulin and glucose, pointing out the remarkable insulin sensitizing effects of MSI-1436 and PTP1B inhibition strategy in EMS affected horses.

## Data availability statement

The original contributions presented in the study are included in the article/supplementary material. Further inquiries can be directed to the corresponding author.

## Ethics statement

The animal study was reviewed and approved by Local Ethics Committee for Animal Experiments in Wroclaw, PAN Ludwik Hirszfeld Institute of Immunology and Experimental Therapy in Wroclaw (Instytut Immunologii i Terapii Doświadczalnej im. Ludwika Hirszfelda PAN we Wrocławiu). (Resolution no. 058/2021/P1 of 23.09.2021 and resolution annex no. 035/2022/NZP/DO of 20.07.2022).

## Author contributions

LB participated in experimental design, coordination, investigation, data analysis and interpretation, manuscript draft writing, editing and final version review. AS-S participated in manuscript draft writing. AP participated in clinical trial, tissue samples collection and manuscript draft writing. MS participated in investigation and manuscript draft writing. MM participated in animals’ management and tissue samples organisation. KM participated in study design, coordination and manuscript writing. All authors contributed to the article and approved the submitted version.

## Glossary

**Table:**

AKT1	Serine/threonine-protein kinase 1
AST	Aspartate aminotransferase
ATF4	Activating Transcription Factor 4
ATF6	Activating Transcription Factor 6
Atg16L1	Autophagy Related 16 Like 1
Atg5	Autophagy Related 5
Atg7	Autophagy Related 7
BAK	Bcl-2 homologous antagonist/killer
BAX	BCL2 Associated X
BCS	Body condition score
BIM	Bcl-2-like protein 11
BiP	Binding immunoglobulin protein
BNIP3	BCL2 and adenovirus E1B 19-kDa-interacting protein 3
BW	Body weight
CEBPA	CCAAT enhancer binding protein
CHOP	CCAAT-enhancer-binding protein homologous protein
CMA	Chaperone-mediated autophagy
CNS	Cresty Neck Score
CTLA-4	Cytotoxic T-Lymphocyte Antigen 4
DGAT1	Diacylglycerol O-Acyltransferase 1
DMEM-LG	Dulbecco’s Modified Eagle Medium with Low glucose
EMS	Equine metabolic syndrome
ER	Endoplasmic reticulum
ERAD	ER-associated degradation
FFAs	Free fatty acids
GGTP	Gamma-glutamyl transpeptidase
GRP94	Glucose-regulated protein 94
HE	Healthy
HepG2	Human hepatocellular carcinoma
HSC70	Heat shock cognate protein 70
HSPA	Heat Shock Protein Family A (Hsp70)
ICOS	Inducible costimulator
IFN-γ	Interferon γ
IL-1β	Interleukin-1β
IL-6	Interleukin-6
IR	Insulin receptor
IR	Insulin resistance
IRS-1	Insulin receptor substrate 1
JAK2	Janus Kinase 2
LC3	Microtubule-associated protein 1A/1B-light chain 3
LMPTP	Low molecular weight phosphotyrosine protein phosphatase
MFN2	Mitofusin 2
mTOR	Mammalian target of rapamycin
NIX	BCL2 Interacting Protein 3 Like
PBMC	Peripheral blood mononuclear cells
PD-1	Programmed death 1
PERK	Protein kinase R (PKR)-like endoplasmic reticulum kinase
PINK1	PTEN-induced kinase 1
PPARA	Peroxisome proliferator-activated receptor alpha
PS	Penicillin –Streptomycin
PTP1B	Protein-tyrosine phosphatase 1B
PTPs	Protein-tyrosine phosphatases
RT-qPCR	Reverse transcription-quantitative polymerase chain reaction
SREBF1	Sterol regulatory element-binding protein
STAT3	Signal transducer and activator of transcription 3
STAT-5	Signal transducer and activator of transcription 5
T2D	Type 2 Diabetes
TGF-β	Transforming growth factor β
TNF-α	Tumor necrosis factor α
TRB3	Tribbles Pseudokinase 3
TRIB3	Tribbles Pseudokinase 3
UPR	Unfolded protein response
Xbp1	X box binding protein-1
ZDF	Zucker diabetic fatty
